# Navigating the genetic landscape of breast cancer in South Africa amidst a developing healthcare system

**DOI:** 10.3389/fgene.2023.1330946

**Published:** 2024-01-08

**Authors:** Jaco Oosthuizen, Nerina C. Van der Merwe, Maritha J. Kotze

**Affiliations:** ^1^ Division of Human Genetics, National Health Laboratory Service, Bloemfontein, South Africa; ^2^ Division of Human Genetics, Faculty of Health Sciences, University of the Free State, Bloemfontein, South Africa; ^3^ Division of Chemical Pathology, Department of Pathology, National Health Laboratory Service, Tygerberg Hospital, Cape Town, South Africa; ^4^ Division of Chemical Pathology, Department of Pathology, Faculty of Medicine and Health Sciences, Stellenbosch University, Cape Town, South Africa

**Keywords:** breast cancer, South Africa, genetic screening, population-directed testing, haplotyping analysis

## Abstract

Breast cancer is a significant global health issue as it represents the leading cause of death in women worldwide. In 2021, the World Health Organization established the Global Breast Cancer Initiative framework with the aim to reduce the breast cancer mortality rate by the year 2040. In countries with developing healthcare systems, such as South Africa, the implementation of first-world technologies has been slow. We provide an overview of the strides taken to improve the cost-effectiveness of genetic service delivery for breast cancer patients in South Africa - from advances in the technology utilized for BRCA founder genotyping to variant screening in moderate-to high-penetrance genes. We furthermore reflect on research undertaken to improve accessibility by means of population-directed point-of-care genetic testing that is ideal for use in a primary healthcare setting. We also report on a pilot study utilizing exome sequencing at the intersection between research and service delivery. Finally, we discuss and conclude on the controversies, research gaps, and future prospects based on the most recent developments in first-world countries that are implementable in developing countries to improve early detection of breast cancer and overall disease management.

## 1 Introduction

Breast cancer is a significant global health issue and a leading cause of death in women worldwide. Therefore, in 2021, the World Health Organization (WHO) established the Global Breast Cancer Initiative (GBCI) framework which sets out to provide a roadmap for reducing the breast cancer mortality rate by 2.5% per year, saving 2.5 million lives by 2040. They aim to enable sustainable health systems in order to deliver breast cancer care in low- and middle-income countries, through proven strategies that are country-specific and resource-appropriate. At its core, the framework recommends that countries focus their actions on developing and improving programs that 1) diagnose at least 60% of breast cancer within the early stage of disease, 2) diagnose breast cancer within 60 days of initial presentation, and 3) manage the disease effectively so that 80% of patients complete their treatment plan (World Health Organization Global Breast Cancer Initiative, 2023).

Various factors contribute to breast cancer risk, prognosis, and management. These range from age, ethnicity, family history, duration of endogenous hormonal exposure, modifiable lifestyle and socio-economic factors, and particularly a genetic predisposition (Daly et al., 2021; Fakhri et al., 2022; Hong and Xu, 2022). To assist healthcare providers in identifying at-risk individuals who may benefit from testing, the National Comprehensive Cancer Network^®^ (NCCN), for example, developed regularly reviewed best practice clinical guidelines for genetic testing and risk assessment (Daly et al., 2023; Gradishar et al., 2023).

Currently, no single genetic service delivery model exists to increase the identification of at-risk individuals in developing healthcare systems. In South Africa, genetic screening services in the public domain focused mainly on germline testing of *BRCA1*/*2* founder variants or limited gene sequencing. Optimal *BRCA1/2* screening was hampered by assay throughput and high costs, which has changed with the introduction of next-generation sequencing (NGS). This resulted in revision of the patient selection criteria, originally limited to a positive family history of breast cancer or an early age of onset. With higher throughput and increased cost effectiveness, the patient selection criteria was broadened. Screening is now also performed for individuals without a family history but diagnosed at an early age (irrespective of tumor characteristics) or diagnosed at a late stage with aggressive disease (SADoH, 2019; van der Merwe et al., 2022a). In contrast, developed countries with fewer financial constraints use extended testing protocols ranging from somatic tumor testing to identify treatment targets, to multi-cancer gene panels or genome sequencing (WGS) that include newly identified, low-penetrance genes for individuals that do not meet the selection criteria.

A significant challenge faced by developing countries implementing advanced genomic technologies is the underestimation of genetic diversity linked to the rapid detection of novel variants, especially in under-sequenced populations (Wong et al., 2020). In this context, the American College of Medical Genetics (ACMG) variant interpretation guidelines offer a standardized framework for variant classification (Richards et al., 2015). This framework standardizes the application of our current knowledge of Mendelian inheritance and monogenic diseases, yet underscores the complexity of variant interpretation. This approach necessitates a multidisciplinary team approach for variant classification that includes a medical geneticist and a genetic counselor. To improve the utilization of the ACMG guidelines, expert variant curation consortia such as the Clinical Genome Resource (ClinGen) group and the Cancer Variant Interpretation Group UK (CanVIG-UK) published guidelines that refine the ACMG recommendations (CanVIG-UK, 2023; Clinicalgenome, 2023). Unfortunately, many laboratories rely predominantly on allele frequency and *in silico*-predicted consequences to determine the functional impact. This complicates classification in developing countries such as South Africa, as many variants are rare, and population-specific genomes underrepresented in international reference or clinical databases. This causes a significant decrease in the ability of laboratories to calculate odds ratios and variant penetrance within a particular disease. Additionally, although *in silico* tools may offer insight into the functional consequence of a variant, these tools do not account for the combined or additive effect of co-segregating variants.

Since identifying *BRCA1* and *BRCA2*, numerous studies have reported on ethnic groups that harbor unique and recurrent pathogenic variants or risk loci (Rebbeck et al., 2018), often assuming founder status based on prevalence alone (Kwon et al., 2022). Moving beyond variant prevalence, haplotype analysis using short tandem repeats (STRs) is the gold standard for confirming founder effects via segmented conservation (haploblocks) of the risk-associated loci. Given the inability of NGS to sequence repeats longer than the amplicon size, studies are increasingly using single nucleotide polymorphisms (SNPs) to infer haplotypes (Tuazon et al., 2020; Boujemaa et al., 2022) through linkage disequilibrium analysis. Therefore, haplotype inference is becoming important for co-segregation analysis of variants of unknown clinical significance (VUS) or variants that may act as modifiers. In this mini-review, we discuss the progress made in breast cancer genetic testing in SA, and future research that may help address current challenges.

## 2 Towards genetic testing in primary healthcare at the point-of-care

**FIGURE 1:**
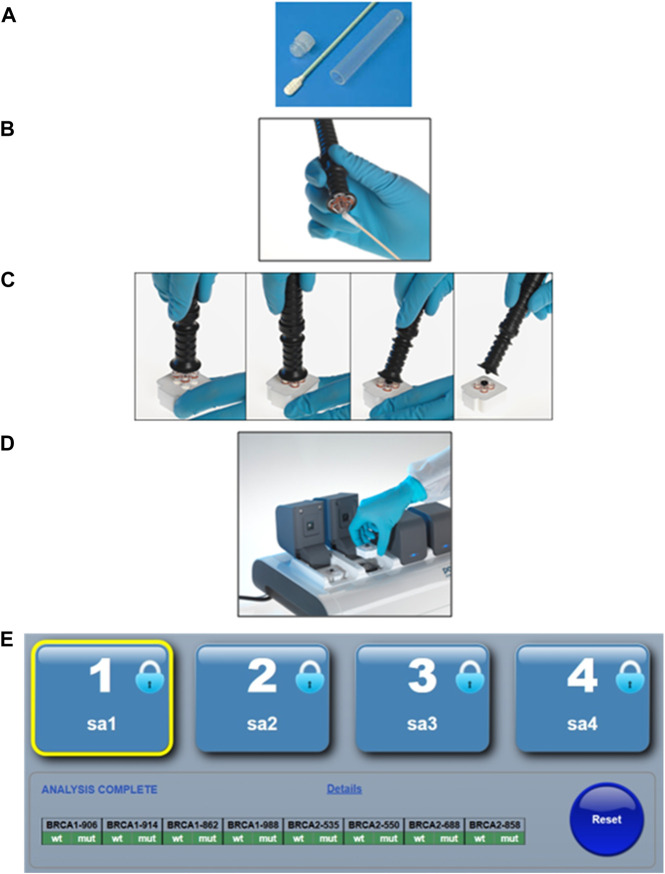
The simplified point-of-care genotyping workflow using the ParaDNA instrument and BRCA founder assay. **(A)** The patient provides a buccal sample by swabbing the inside of each cheek 10–15 times. **(B)** The operator transfers the sample to a sample collector. **(C)** The sample collector is inserted into the reaction plate, and the handle is snapped off. **(D)** The reaction plate is then inserted into the PCR instrument, where after DNA amplification is performed through thermocycling and variant detection through end-point genotyping. **(E)** The software analyses the melt peaks and reports the genotypes in a simplified text-based summary output. Permission was requested and granted from LGC Ltd to present the workflow of the POCT as presented in Figure 1.

The South Africa Department of Health (DoH) clinical guidelines for breast cancer control and management released in 2019, outlined various goals and strategic objectives aimed at reducing the burden of breast cancer by 2030. The first key area focuses on prevention, early detection, screening, and genetic assessment with referral to specialized breast units available at South African tertiary hospitals. The South Africa DoH’s envisioned goal is to transform regional primary care facilities into one-stop diagnostic clinics, where patients can be assessed and, if necessary, be directed to the appropriate specialized referral pathway following a single visit (SADoH, 2019). Given the criteria for these one-stop diagnostic clinics, they are well-suited for simplified, first-tier population-directed genetic screening to identify well-established founder variants previously detected for South Africa in the *BRCA1* and *BRCA2* genes (Figure 1).

Harnessing the potential of population- or disease-directed genotyping assays has been proposed as a suitable approach to implementing genetic testing in primary healthcare settings. This is ideally suited for recurrent variants for which their associated risks and treatment or management plans are well-defined. Molecular point-of-care testing (POCT) offers rapid turnaround times for founder/recurrent variant screening when compared to standard laboratory test methodologies, with the potential to guide timely treatment and improve clinical and economic outcomes. Portable devices that are user-friendly enable sufficiently trained healthcare workers to bring genetic testing to their patients, significantly reducing the time it currently takes for a clinician to make a medical decision. One such example is the potential use of a newly designed population-directed POCT genotyping assay screening for common *BRCA1/2* South African founder variants, proposed as a first-tier test in all breast and ovarian cancer patients (Figure 1). These rapid multiplexed *BRCA1/2* POCT assays are more cost-effective than their laboratory-based counterparts or the routinely used NGS assays. However, the global acceptance of genetic POCT has been slow, with many arguing that the risk remains too high when conducted without proper genetic counseling. A poll completed during a workshop hosted as part of the local biennial Southern African Society for Human Genetics (SASHG) conference revealed that 94% of the members attending approved the use of POCT as a cost-effective first-tier test if accompanied by genetic counseling (Mampunye et al., 2021; Oosthuizen et al., 2021). As less than 10% of South Africa’s need for clinical genetic services is currently being met, the demand for skilled professionals who can provide and interpret clinically actionable genetic information will likely be met in two ways, namely, by upskilling primary care health professionals including general practitioners and nurses, and upscaling current training of GCs and medical geneticists (van der Merwe et al., 2022b).

With the advent of POCT, the involvement of a genetic counselor or trained genetics professional is important given the potential psychosocial risks associated with the receipt of immediate results associated with medical or familial implications. The benefits *versus* the risks or potential harm of receiving an immediate genetic result are yet to be properly assessed in various testing scenarios in the local setting. Rayes et al. (2019) contended that while in-person genetic counseling is likely to remain standard practice in large cancer centers and academic institutions, alternative service delivery models for genetic counseling must be explored. The authors stated that an optimal balance of clinical quality with increased access and financial sustainability in the setting of population screening for hereditary breast and ovarian cancer (HBOC) and other cancer syndromes needs to be found. We share this sentiment as innovative service delivery models, including telephonic or online genetic counseling, could be essential to decreasing barriers to genetic testing, particularly amongst underserved and under-resourced populations. We suggest that rapid genotyping or POCT may be feasible in primary healthcare facilities provided that the clinical and counseling service delivery models have been optimized for the specific healthcare system. This strategy may enable the identification of individuals at higher risk based on their demographic characteristics as well as provide genetic testing to those who might otherwise not have had access to genetic testing for HBOC.

## 3 Screening for all: gene panels *versus* the exome sequencing

The Breast Cancer Association Consortium (BCAC) and Ovarian Cancer Association Consortium (OCAC) have identified a list of 34 loci significantly associated with breast cancer risk. In 2021, BCAC published the summary results of the BRIDGES sequencing project, an international effort aimed at investigating the variant spectrum of 34 breast cancer susceptibility genes previously identified through GWAS. The primary goal was to sequence the risk loci in a case-control cohort of approximately 113,000 females to determine the spectrum of truncating and missense variants within these genes and estimate odds ratios for their overall breast cancer risk as well as correlation to specific tumor subtypes. Furthermore, the study provided insights into the association of these genes with estrogen receptor-positive and estrogen receptor-negative breast cancer (Dorling et al., 2021).

**FIGURE 2:**
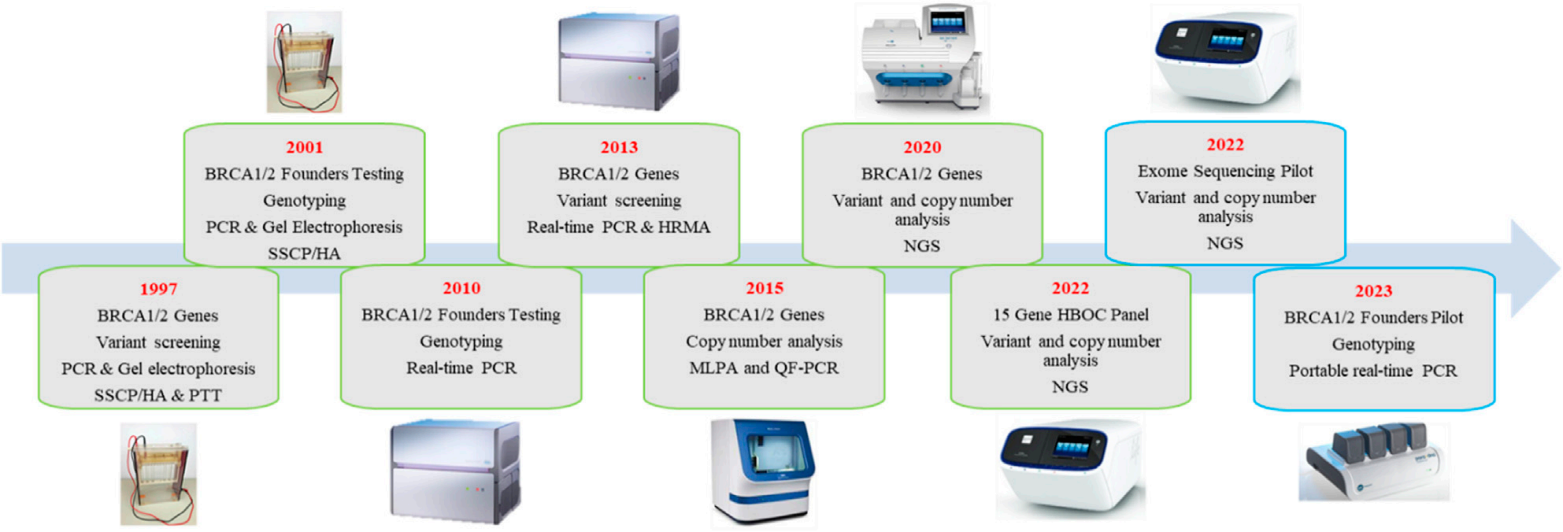
Flow chart depicting the methodologies utilized for breast cancer genetic testing in the public domain in South Africa over the last two decades. Text boxes outlined in green represent methodologies which were implemented for routine diagnostic screening, while blue boxes indicate methodologies currently used in research.

In South Africa, genetic testing within the public domain heavily relies on international evidence-based guidelines and consortia, such as the NCCN and BCAC. The adoption of first-world technologies and more flexible screening guidelines promotes equity and equality in genetic services. However, its full application remains a challenge in countries with significant budget constraints. In 2022, we published a follow-up study on the public perspective of breast cancer genetic testing in South Africa (van der Merwe et al., 2022c). We reported on the implementation of a multigene NGS panel after more than two decades of *BRCA1* and *BRCA2*-only testing. Figure 2 illustrates the evolution of testing methodologies over the past 20 years together with current research projects that aim to improve the cost-effectiveness of genetic testing. The use of multigene panel testing not only extended beyond *BRCA1/2* but also offered the added benefit of identifying genetic variants in genes with pharmacogenetic relevance. Patients harboring pathogenic variants in these genes might benefit from treatment with poly (ADP-ribose) inhibitors, though they are currently costly and unavailable in the public sector of South Africa. Fortunately, the 15-gene panel could be offered at the same cost as the previous 2-gene *BRCA1/2* assay due to the reduction in NGS costs worldwide. The distribution of variants in the detected genes was comparable to those of the BCAC BRIDGES project and NCCN guidelines, highlighting the effectiveness of established selection criteria used when screening for HBOC familial risk. The South African study further explored the additional clinical value of exome sequencing at the intersection of diagnostic service delivery and research. Limiting variant interpretation to an 84-gene hereditary cancer virtual panel reduced the time spent on variant interpretation involving a high rate of VUSes, and minimized the risk of incidental findings (van der Merwe et al., 2022a).

The additional application of pathology-supported genetic testing (PSGT) furthermore enhanced diagnostic accuracy (van der Merwe et al., 2017; Okunola et al., 2023). Examining tumor characteristics alongside genetic analysis aids in determining therapeutic strategies and understanding the genetic basis of cancer. Deciding which and how many genes should be evaluated, however, remains a challenge. This can partially be attributed to differences observed with regard to the penetrance of pathogenic variants observed even in the well-known high-penetrant class. For example, some truncating variants within BRCA1 and BRCA2 exhibit a lower penetrance than expected, leading to a lower lifetime cancer risk. Understanding the genomic structure and protein consequence of co-segregating variants, the spectrum of these variants, and the specific haplotype penetrance, has the potential to increase the accuracy of risk assessments, incidence reporting, and clinical decision-making.

## 4 Haplotype inference: fine mapping risk loci in routine diagnostics

The study of co-segregating variants and regulatory elements associated with risk loci in breast cancer has internationally become an attractive research topic. The cumulative effect of risk variants across the genome is expressed as a polygenic risk score (PRS). In 2023, Roberts and others (2023) reported that over 30% of breast cancer heritability may be explained by low-risk variants previously identified through GWAS. Knowing the genomic spectrum of recurrent variants unique to a particular population increases the discriminatory power of the tool and enables appropriate calibration of the PRS (Roberts et al., 2023). Initiatives such as the Three Million African Genome (3MAG) project envisioned by the African Society of Human Genetics (AfSHG) and the 100K Human Genome Project planned by the South African Medical Research Council (SAMRC), may in the future enable the application of PRS in developing African countries. While the clinical utility of PRS may only be realized in years to come in South Africa, sufficient SNP data is routinely generated for the high and moderate-penetrance breast cancer genes to perform haplotype inference of the disease-associated loci.

In a study by Tuazon et al. (2020), the authors used SNPs to estimate the haplotype diversity of a *BRCA1* pathogenic variant. The group had phased SNPs in *BRCA1* and estimated the haplotype age. They concluded that genome-wide SNP data can be used to assess relatedness between individuals from different population groups that harbor the same pathogenic variant. Oosthuizen *et al.* (2021) showed that by using NGS-based SNP data with a population allele frequency greater than 1%, ancestral haplotypes could be inferred for *BRCA2*. These haplotypes represented 96% of the *BRCA2* haplotype diversity in South Africa and could be used to confirm local founder effects (Oosthuizen et al., 2021). Although this approach could infer ancestral haplotypes, it could not significantly associate novel rare variants with a rare, family-specific haplotype. As a next step, Boujemaa *et al.* (2022) characterized the haplotypes of nine high and moderate-penetrance breast cancer susceptibility genes using SNPs from GWAS studies. Their study revealed distinct linkage disequilibrium patterns and identified putative functional SNPs that may be used to develop PRS.

In 2021, Ruiz De Garibay and others (2021) reported a common intron 10 *BRCA1* gene variant (rs5820483, NC_000017.11:g.43095106_43095108dup) that was proven to induce the alternative splicing of exon 11, resulting in an altered expression of a BRCA1 isoform. It was suggested that perturbation of *BRCA1* exon 11 splicing modifies the breast cancer risk conferred by pathogenic variants in the gene. This intronic variant is rarely reported in conventional screening assays that focus on coding variants only, due to its intronic gene location. Ruiz De Garibay et al. (2021) used linkage disequilibrium analysis to infer *BRCA1* haplotypes of pathogenic variants and confirmed the presence of founder groups. Another controversial variant frequently under review for more than 20 years since the discovery of *BRCA2* that requires haplotype analysis, is the C-terminal K3326X variant in exon 27. Mazoyer *et al.* (1996) reported the variant as a polymorphic stop-codon due to its co-segregation with another pathogenic variant. Based on this observation, the variant was later classified as benign, with some studies reporting that its association with breast cancer risk was overestimated. Since then, several studies have reported alternate haplotypes in which a co-segregating pathogenic variant was absent (Higgs et al., 2015; Roberts et al., 2023). The latest evidence indicates are that this variant may have therapeutic value, and although the associated breast cancer risk is low, the odds ratios are significant for several other cancer types (Valiakhmetova et al., 2020; Baughan and Tainsky, 2021).

This serves a prime example in which haplotype analysis could play a significant role in understanding the co-segregation of genetic variants and their phenotypic penetrance. In addition to leveraging the throughput of population-based genotyping assays and NGS, linkage disequilibrium analysis in the routine diagnostic workflow could prevent potential misclassification of pathogenic variants in instances where the suspected variant does not necessarily segregate with the disease.

## 5 Discussion and future directions

### 5.1 Controversies

The selection of optimal test methods for breast cancer population-based testing presents one of the main controversies under current ethical debate. Genotyping is cost-effective but may miss rare variants, especially in diverse and understudied populations where founder variants contribute to <10% of the total mutation frequency (Foulkes et al., 2016; Makhetha et al., 2023), preventing fair access to personalized treatment plans. However, a major benefit of a population-directed cost-effective portable POC genotyping assay together with innovative/alternative counseling models, is that it could enable screening in rural areas, which may increase access to genetic testing and improve primary care and cancer awareness in developing healthcare systems. NGS offers a more comprehensive approach but requires specialized staff and infrastructure, incurring higher costs. Higher costs may further reduce access to testing and may increase post-test distress when a VUS is reported, thereby resulting in exome sequencing being a future option for routine diagnostics in the public domain in developing countries.

### 5.2 Research gaps

A contentious issue is the management of variants of variable clinical significance and low penetrant genes. Their clinical significance remains a subject of ongoing research, making it difficult to develop clear guidelines for its widespread inclusion in testing. An example here is a variant that is the minor allele in one population but a major allele in another. There is a pressing need for research in developing countries to understand the prevalence and co-segregation of genetic variants within their respective populations. Additionally, there is a lack of consensus regarding the use of linkage disequilibrium to determine co-segregation and infer loci-specific haplotypes in the routine diagnostic setting due to the lack of large population datasets and the underestimation of the genomic diversity of underrepresented population groups.

### 5.3 Future developments

Future developments in genetic testing for hereditary breast cancer will likely involve the integration of artificial intelligence and machine learning to improve variant interpretation where there is a shortage of specialized clinical and molecular staff. The introduction of long-range sequencing can overcome the bioinformatics challenges of haplotype analysis and phasing when the cohort is too small and statistically insignificant. Tailoring genetic testing strategies to specific populations and understanding the interplay of multiple genetic factors, will be crucial in developing more precise risk assessment models. Furthermore, international collaborations and data sharing for PSGT are vital for expanding our understanding of genetic variants and their clinical implications when using exome/genome sequencing in the routine diagnostic setting.
